# Supplementary material for: Amyloid β1–42 Oligomers Induce Galectin–1S8 O–GlcNAcylation Leading to Microglia Migration
Source: Cells. 2023 Jul 17;12(14):1876. doi: 10.3390/cells12141876 (PMC10378097; doi:10.3390/cells12141876)
Supplement: Supplementary file 1 [file cells-12-01876-s001.zip › cells-2430029-supplementary.pdf]

Supplementary data

Table S1. O-GlcNAcylated proteins exclusively identified in the Aβ_{1-42} oligomers-stimulated microglia.

UniProt KB Reference	Identified Protein	Gene
**Q13885;Q9BVA1**	Tubulin beta-2A chain; Tubulin beta-2B chain	TBB2A; TBB2B
**Q16576; Q16576-2**	Histone-binding protein RBBP7	RBBP7
**Q8IW75**	Serpin A12	SERPINA12
**P14618; P14618-3; P14618-2**	Pyruvate kinase PKM	PKM
**P14625**	Endoplasmin	HSP90B1
**P60842-2; P60842; Q14240; Q14240-2**	Eukaryotic initiation factor 4A-I	EIF4A1
**Q12907**	Vesicular integral-membrane protein VIP36	LMAN2
**O15118;O15118-2**	Niemann-Pick C1 protein	NPC1
**P09382**	Galectin-1	LGALS1
**P17050**	Alpha-N-acetylgalactosaminidase	NAGA
**P43121; P43121-2**	Cell surface glycoprotein MUC18	MCAM
**P52272-2; P52272**	Heterogeneous nuclear ribonucleoprotein M	HNRNPM
**P60174-1; P60174; P60174-4**	Triosephosphate isomerase	TPI1
**Q13867**	Bleomycin hydrolase	BLMH
**Q6EMK4**	Vasorin	VASN
**Q6P4E1-2; Q6P4E1-5; Q6P4E1; Q6P4E1-4; Q6P4E1-3**	Protein CASC4	CASC4
**Q6ZRP7**	Sulfhydryl oxidase 2	QSOX2
**Q71U36-2; Q71U36; P0DPH8; P0DPH7; Q6PEY2; P0DPH7-2**	Tubulin alpha-1A chain;Tubulin alpha-3E chain	TUBA1A;TUBA3E
**Q96KR1**	Zinc finger RNA-binding protein	ZFR
**Q9H0E3; Q9H0E3-3; Q9H0E3-2**	Histone deacetylase complex subunit SAP130	SAP130
**O43813**	LanC-like protein 1	LANCL1
**P30041**	Peroxiredoxin-6	PRDX6

**P10909-3; P10909-4; P10909; P10909-5; P10909-2**	Clusterin; Clusterin beta chain; Clusterin alpha chain	CLU
**P15289-2; P15289**	Arylsulfatase A; Arylsulfatase A component B; Arylsulfatase A component C	ARSA
**Q5TFQ8; P78324-4; P78324; P78324-2**	Signal-regulatory protein beta-1 isoform 3; Tyrosine-protein phosphatase non-receptor type substrate 1	SIRPB1;SIRPA
**Q92484-2; Q92484**	Acid sphingomyelinase-like phosphodiesterase 3a	SMPDL3A
**Q92796**	Disks large homolog 3	DLG3
**Q96JA1-2; Q96JA1**	Leucine-rich repeats and immunoglobulin-like domains protein 1	LRIG1
**P30101**	Protein disulfide-isomerase A3	PDIA3
**P61224-2; P61224-4; P61224-3; P62834; P61224**	Ras-related protein Rap-1b; Ras-related protein Rap-1A	RAP1B;RAP1A
**P62249**	40S ribosomal protein S16	RPS16
**Q14697; Q14697-2**	Neutral alpha-glucosidase AB	GANAB
**P05141; P12235; Q9H0C2**	ADP/ATP translocase 2; ADP/ATP translocase 2, N-terminally processed; ADP/ATP translocase 1	SLC25A5; SLC25A4
**P42892-3; P42892-2; P42892-4; P42892**	Endothelin-converting enzyme 1	ECE1
**P51116; P51114-3; P51114-2; P51114; Q06787-8; Q06787-6; Q06787-2; Q06787-4; Q06787-5; Q06787-9; Q06787-7; Q06787-3; Q06787**	Fragile X mental retardation syndrome-related protein 2; Fragile X mental retardation syndrome-related protein 1	FXR2; FXR1
**P67809; P16989-2; P16989-3; Q9Y2T7; P16989**	Nuclease-sensitive element-binding protein 1	YBX1
**Q99623-2; Q99623**	Prohibitin-2	PHB2
**Q9Y2E5-2; Q9Y2E5**	Epididymis-specific alpha-mannosidase	MAN2B2
**P18124**	60S ribosomal protein L7	RPL7

**P48643-2; P48643**	T-complex protein 1 subunit epsilon	TCPE
**Q15365; P57721-2; P57721-3; P57721-5; P57721-4; P57721**	Poly(rC)-binding protein 1; Poly(rC)-binding protein 3	PCBP1; PCBP3
**P33402; P33402-3; P33402-2**	Guanylate cyclase soluble subunit alpha-2	GUCY1A2
**Q04118**	Basic salivary proline-rich protein 3	PRB3
**Q9H6L2-2**	Transmembrane protein 231	TMEM231
**Q9ULF5**	Zinc transporter ZIP10	SLC39A10
